# 1-Methane­sulfonyl-1*H*-1,2,3-benzotriazole

**DOI:** 10.1107/S1600536810040778

**Published:** 2010-10-20

**Authors:** Petr Štěpnička, Hana Solařová, Ivana Císařová

**Affiliations:** a Department of Inorganic Chemistry, Faculty of Science, Charles University in Prague; Hlavova 2030, 12840 Prague 2, Czech Republic

## Abstract

The mol­ecular geometry of the title compound, C_7_H_7_N_3_O_2_S, does not differ much from that of the previously reported 4-toluene­sulfonyl analogue. Unlike the latter compound, however, mol­ecules of the title compound associate primarily *via* π–π stacking inter­actions of their benzene rings [centroid–centroid distance = 3.5865 (8) Å], forming columnar stacks along the crystallographic 2_1_ axes. These stacks are inter­connected *via* weak C—H⋯O and C—H⋯N hydrogen bonds.

## Related literature

For crystal structure of 1-(*p*-toluene­sulfon­yl)-1*H*-1,2,3-benzotriazole, see: Rodríguez *et al.* (2005 ▶). For the preparation of the title compound and examples of its synthetic use, see: Katritzky *et al.* (1992 ▶, 2000 ▶).
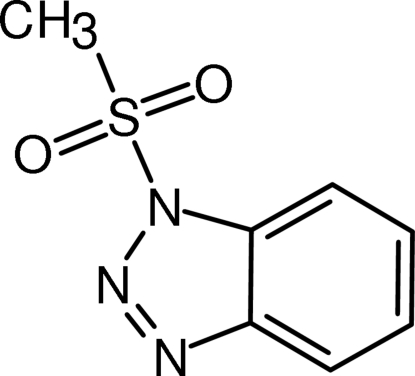


## Experimental

### 

#### Crystal data

C_7_H_7_N_3_O_2_S
*M* *_r_* = 197.22
Monoclinic, 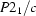

*a* = 9.3685 (3) Å
*b* = 7.0627 (2) Å
*c* = 12.4994 (3) Å
β = 92.984 (2)°
*V* = 825.93 (4) Å^3^
*Z* = 4
Mo *K*α radiation
μ = 0.36 mm^−1^
*T* = 150 K
0.50 × 0.30 × 0.25 mm

#### Data collection

Nonius KappaCCD diffractometer
14989 measured reflections
1886 independent reflections
1674 reflections with *I* > 2σ(*I*)
*R*_int_ = 0.023

#### Refinement

*R*[*F* ^2^ > 2σ(*F* ^2^)] = 0.030
*wR*(*F* ^2^) = 0.082
*S* = 1.09
1886 reflections
119 parameters
H-atom parameters constrained
Δρ_max_ = 0.29 e Å^−3^
Δρ_min_ = −0.42 e Å^−3^

### 

Data collection: *COLLECT* (Nonius, 2000 ▶); cell refinement: *SCALEPACK* (Otwinowski & Minor, 1997 ▶); data reduction: *DENZO* (Otwinowski & Minor, 1997 ▶) and *SCALEPACK*; program(s) used to solve structure: *SIR97* (Altomare *et al.*, 1999) ▶; program(s) used to refine structure: *SHELXL97* (Sheldrick, 2008 ▶); molecular graphics: *PLATON* (Spek, 2009 ▶); software used to prepare material for publication: *PLATON*.

## Supplementary Material

Crystal structure: contains datablocks I, global. DOI: 10.1107/S1600536810040778/im2237sup1.cif

Structure factors: contains datablocks I. DOI: 10.1107/S1600536810040778/im2237Isup2.hkl

Additional supplementary materials:  crystallographic information; 3D view; checkCIF report

## Figures and Tables

**Table 1:** Hydrogen-bond geometry (Å, °)

*D*—H⋯*A*	*D*—H	H⋯*A*	*D*⋯*A*	*D*—H⋯*A*
C5—H5⋯O2^i^	0.93	2.55	3.270 (2)	135
C6—H6⋯O1^ii^	0.93	2.55	3.451 (2)	164
C8—H8*B*⋯N3^iii^	0.96	2.61	3.446 (2)	145
C8—H8*C*⋯O2^iv^	0.96	2.40	3.325 (2)	161

Symmetry codes: (i) 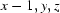; (ii) 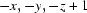; (iii) 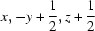; (iv) 
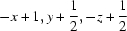
.

**Fig. 1.**
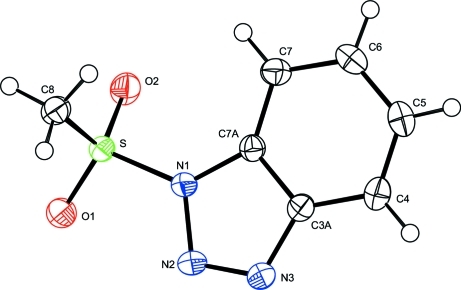
Molecular structure of the title compound showing displacement ellipsoids for the non-H atoms at the 50% probability level. Hydrogen atoms are presented as spheres with arbitrary radii.

**Fig. 2.**
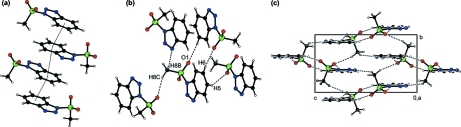
(a) Section of columnar stacks connected by π–π interactions of the benzene rings [Cg···Cg = 3.5865 (8) Å]. (b) H-bond interactions generated by the molecules of the title compounds. (c) View of the unit cell along the crystallographic a axis.

## supplementary crystallographic information

### Comment

The title compound, 1-(methanesulfonyl)-1*H*-1,2,3-benzotriazole, is a useful and readily accessible organic reagent, acting as a convenient source of the benzotriazolyl anion. For instance, it reacts smoothly with carboxylic acids in the presence of a base to afford the corresponding 1-acyl-1*H*-1,2,3-benzotriazoles, which can be subsequently converted to amides in typically good yields (Katritzky *et al.*, 2000).

The molecular structure of the title compound (Fig. 1) is rather unexceptional, particularly in view of the geometric data reported earlier for the related 1*H*-1,2,3-benzotriazole derivative, 1-(*p*-toluenesulfonyl)-1*H*-1,2,3-benzotriazole (Rodríguez *et al.*, 2005). The N—N bonds within the triazole ring clearly maintain their localized character (*cf.* N1—N2 = 1.389 (2) Å, N3—N2 = 1.288 (2) Å), which is, however, not reflected in the adjacent bonds. The lengths of the remaining in-ring bonds, N1—C7A, C7A—C3A and C3A—N3, differ by less than *ca* 0.006 Å, while all in-ring angles span a range of 103.5 (1)–109.9 (1) °. On the other hand, the variation in the analogous parameters describing the geometry of the annelating benzene ring is less pronounced (*cf.* C—C = 1.371 (2)–1.408 (2) Å, C—C—C = 115.5 (1)–122.7 (1) °).

The methanesulfonyl group binds to the triazole ring somewhat unsymmetrically, which is best demonstrated by the difference in the S—N1—N2 (120.12 (9) °) and S—N1—C7A (129.8 (1) °) angles. Moreover, it is angularly distorted: The bond angles around sulfur span a range of 103.43 (6)–120.31 (7) ° with the N1—S—C8 and O1—S—O2 angles being the lower and upper limit, respectively. The remaining angles do not differ much in the pairs: N1—S—O(1/2) (*ca* 105 °, difference *ca* 0.4 °), C8—S1—O(1/2) (*ca* 110 °, difference *ca* 1.2 °). Indeed, such a variation in bond angles corresponds with distances to the sulfur atom (S—O1 1.425 (1), S—O2 1.419 (1), S—N1 1.692 (1), S—C8 1.744 (2) Å) as the most acute angle is associated with the shortest bonds.

In the crystal, molecules of the title compound assemble *via*π–π stacking interactions of their benzene rings (Fig. 2a). Since this interaction involves molecules related by the crystallographic 2_1_ screw axes, it results in the formation of infinite columnar stacks in the direction of the crystallographic *b* axis. It is worth pointing out that the observed separation of the ring centroids [*Cg*···*Cg* (-*x*, 1/2 + *y*, 1/2-*z;* 3.5865 (8) Å] is slightly shorter than that reported for α-graphite (*ca* 3.65 Å), where, however, the rings are slipped by *ca* 1.42 Å. Finally, the neighboring columnar stacks are interlinked by soft C—H···O and C—H···N hydrogen bonds (Table 1) into a complicated three-dimensional array (Figs. 2b and 2c).

### Experimental

The title compound was synthesized from 1*H*-1,2,3-benzotriazole and methanesulfonyl chloride as described in the literature with a yield of 91% (Katritzky *et al.*, 2000). Crystals suitable for X-ray diffraction analysis were obtained by crystallization from warm benzene.

### Refinement

H-atoms were included in calculated positions and refined as riding atoms with fixed C—H distances [C—H = 0.96 Å for CH_3_, and 0.93 Å for aromatic CH] and *U*_iso_(H) assigned to 1.5*U*_eq_(C) (CH_3_) or 1.2*U*_eq_(C) (aromatic CH) of their bonding carbon atom.

### Crystal data

**Table:**

C_7_H_7_N_3_O_2_S	*F*(000) = 408
*M**_r_* = 197.22	*D*_x_ = 1.586 Mg m^−^^3^
Monoclinic, *P*2_1_/*c*	Mo *K*α radiation, λ = 0.71073 Å
Hall symbol: -P 2ybc	Cell parameters from 2027 reflections
*a* = 9.3685 (3) Å	θ = 1.0–27.5°
*b* = 7.0627 (2) Å	µ = 0.36 mm^−^^1^
*c* = 12.4994 (3) Å	*T* = 150 K
β = 92.984 (2)°	Block, colourless
*V* = 825.93 (4) Å^3^	0.50 × 0.30 × 0.25 mm
*Z* = 4	

### Data collection

**Table:**

Nonius KappaCCD diffractometer	1674 reflections with *I* > 2σ(*I*)
Radiation source: fine-focus sealed tube	*R*_int_ = 0.023
horizontally mounted graphite crystal	θ_max_ = 27.5°, θ_min_ = 2.2°
Detector resolution: 9.091 pixels mm^-1^	*h* = −12→12
ω and π scans to fill the Ewald sphere	*k* = −9→9
14989 measured reflections	*l* = −16→16
1886 independent reflections	

### Refinement

**Table:**

Refinement on *F*^2^	Primary atom site location: structure-invariant direct methods
Least-squares matrix: full	Secondary atom site location: difference Fourier map
*R*[*F*^2^ > 2σ(*F*^2^)] = 0.030	Hydrogen site location: inferred from neighbouring sites
*wR*(*F*^2^) = 0.082	H-atom parameters constrained
*S* = 1.09	*w* = 1/[σ^2^(*F*_o_^2^) + (0.0431*P*)^2^ + 0.2983*P*] where *P* = (*F*_o_^2^ + 2*F*_c_^2^)/3
1886 reflections	(Δ/σ)_max_ < 0.001
119 parameters	Δρ_max_ = 0.29 e Å^−^^3^
0 restraints	Δρ_min_ = −0.42 e Å^−^^3^

### Special details

**Table:**

Geometry. All e.s.d.'s (except the e.s.d. in the dihedral angle between two least-squares planes) are estimated using the full covariance matrix. The cell e.s.d.'s are taken into account individually in the estimation of e.s.d.'s in distances, angles and torsion angles; correlations between e.s.d.'s in cell parameters are only used when they are defined by crystal symmetry. An approximate (isotropic) treatment of cell e.s.d.'s is used for estimating e.s.d.'s involving least-squares planes.
Refinement. Refinement of *F*^2^ against all diffractions. The weighted *R*-factor *wR* and goodness of fit *S* are based on *F*^2^, conventional *R*-factors *R* are based on *F*, with *F* set to zero for negative *F*^2^. The threshold expression of *F*^2^ > 2σ(*F*^2^) is used only for calculating *R*-factors(gt) *etc*. and is not relevant to the choice of reflections for refinement. *R*-factors based on *F*^2^ are statistically about twice as large as those based on *F*, and *R*- factors based on all data will be even larger.

### Fractional atomic coordinates and isotropic or equivalent isotropic displacement parameters (Å^2^)

**Table:**

	*x*	*y*	*z*	*U*_iso_*/*U*_eq_	
S	0.39056 (3)	0.07483 (5)	0.36695 (2)	0.02263 (12)	
O1	0.32920 (11)	−0.04099 (16)	0.44586 (8)	0.0318 (3)	
O2	0.51042 (11)	0.01011 (18)	0.31253 (8)	0.0337 (3)	
N1	0.25880 (13)	0.10614 (17)	0.27080 (9)	0.0236 (3)	
N2	0.29032 (13)	0.10541 (18)	0.16344 (9)	0.0274 (3)	
N3	0.17185 (13)	0.11648 (19)	0.10673 (9)	0.0289 (3)	
C3A	0.05897 (15)	0.1243 (2)	0.17395 (11)	0.0233 (3)	
C4	−0.08866 (16)	0.1336 (2)	0.14889 (12)	0.0290 (3)	
H4	−0.1253	0.1381	0.0784	0.035*	
C5	−0.17615 (16)	0.1359 (2)	0.23349 (12)	0.0295 (3)	
H5	−0.2746	0.1413	0.2200	0.035*	
C6	−0.12050 (16)	0.1302 (2)	0.34029 (12)	0.0290 (3)	
H6	−0.1837	0.1318	0.3953	0.035*	
C7	0.02458 (16)	0.1222 (2)	0.36641 (11)	0.0273 (3)	
H7	0.0611	0.1199	0.4370	0.033*	
C7A	0.11237 (14)	0.11801 (19)	0.27980 (11)	0.0218 (3)	
C8	0.41970 (16)	0.3025 (2)	0.41766 (11)	0.0277 (3)	
H8A	0.4901	0.2977	0.4762	0.042*	
H8B	0.3319	0.3521	0.4424	0.042*	
H8C	0.4531	0.3828	0.3623	0.042*	

### Atomic displacement parameters (Å^2^)

**Table:**

	*U*^11^	*U*^22^	*U*^33^	*U*^12^	*U*^13^	*U*^23^
S	0.02097 (19)	0.0276 (2)	0.01921 (18)	0.00087 (12)	0.00002 (12)	0.00117 (12)
O1	0.0327 (6)	0.0361 (6)	0.0263 (5)	−0.0063 (5)	−0.0026 (4)	0.0100 (4)
O2	0.0245 (5)	0.0461 (7)	0.0306 (5)	0.0088 (5)	0.0010 (4)	−0.0071 (5)
N1	0.0209 (6)	0.0317 (6)	0.0183 (5)	0.0002 (5)	0.0016 (4)	0.0007 (4)
N2	0.0275 (6)	0.0369 (7)	0.0180 (5)	−0.0017 (5)	0.0030 (4)	0.0007 (5)
N3	0.0264 (6)	0.0402 (7)	0.0201 (6)	−0.0023 (5)	0.0002 (5)	0.0017 (5)
C3A	0.0252 (7)	0.0222 (7)	0.0223 (6)	−0.0016 (5)	−0.0002 (5)	0.0008 (5)
C4	0.0266 (7)	0.0293 (7)	0.0304 (7)	−0.0015 (6)	−0.0052 (6)	0.0009 (6)
C5	0.0220 (7)	0.0251 (7)	0.0413 (8)	−0.0012 (6)	0.0005 (6)	0.0008 (6)
C6	0.0263 (7)	0.0262 (7)	0.0352 (8)	0.0000 (6)	0.0100 (6)	0.0008 (6)
C7	0.0290 (7)	0.0294 (7)	0.0238 (7)	0.0010 (6)	0.0038 (5)	0.0010 (6)
C7A	0.0212 (6)	0.0217 (6)	0.0225 (6)	0.0001 (5)	0.0005 (5)	0.0006 (5)
C8	0.0301 (7)	0.0290 (7)	0.0239 (6)	−0.0017 (6)	0.0001 (5)	−0.0003 (6)

### Geometric parameters (Å, °)

**Table:**

S—O2	1.4185 (10)	C4—H4	0.9300
S—O1	1.4254 (11)	C5—C6	1.408 (2)
S—N1	1.6919 (12)	C5—H5	0.9300
S—C8	1.7444 (15)	C6—C7	1.382 (2)
N1—C7A	1.3848 (17)	C6—H6	0.9300
N1—N2	1.3890 (16)	C7—C7A	1.3936 (19)
N2—N3	1.2878 (17)	C7—H7	0.9300
N3—C3A	1.3856 (18)	C8—H8A	0.9600
C3A—C7A	1.3905 (18)	C8—H8B	0.9600
C3A—C4	1.4037 (19)	C8—H8C	0.9600
C4—C5	1.371 (2)		
			
O2—S—O1	120.31 (7)	C4—C5—H5	119.2
O2—S—N1	105.55 (6)	C6—C5—H5	119.2
O1—S—N1	105.13 (6)	C7—C6—C5	122.41 (14)
O2—S—C8	111.01 (7)	C7—C6—H6	118.8
O1—S—C8	109.79 (7)	C5—C6—H6	118.8
N1—S—C8	103.43 (6)	C6—C7—C7A	115.48 (13)
C7A—N1—N2	109.87 (11)	C6—C7—H7	122.3
C7A—N1—S	129.75 (9)	C7A—C7—H7	122.3
N2—N1—S	120.12 (9)	N1—C7A—C3A	103.50 (11)
N3—N2—N1	108.12 (11)	N1—C7A—C7	133.75 (13)
N2—N3—C3A	109.37 (11)	C3A—C7A—C7	122.74 (13)
N3—C3A—C7A	109.12 (12)	S—C8—H8A	109.5
N3—C3A—C4	129.85 (13)	S—C8—H8B	109.5
C7A—C3A—C4	121.02 (13)	H8A—C8—H8B	109.5
C5—C4—C3A	116.74 (13)	S—C8—H8C	109.5
C5—C4—H4	121.6	H8A—C8—H8C	109.5
C3A—C4—H4	121.6	H8B—C8—H8C	109.5
C4—C5—C6	121.60 (14)		

### Hydrogen-bond geometry (Å, °)

**Table:**

*D*—H···*A*	*D*—H	H···*A*	*D*···*A*	*D*—H···*A*
C5—H5···O2^i^	0.93	2.55	3.270 (2)	135
C6—H6···O1^ii^	0.93	2.55	3.451 (2)	164
C8—H8B···N3^iii^	0.96	2.61	3.446 (2)	145
C8—H8C···O2^iv^	0.96	2.40	3.325 (2)	161

Symmetry codes: (i) *x*−1, *y*, *z*; (ii) −*x*, −*y*, −*z*+1; (iii) *x*, −*y*+1/2, *z*+1/2; (iv) −*x*+1, *y*+1/2, −*z*+1/2.
